# Culture and characterization of canine and feline corneal epithelial organoids: A new tool for the study and treatment of corneal diseases

**DOI:** 10.3389/fvets.2022.1050467

**Published:** 2022-11-04

**Authors:** Leila Bedos, Hannah Wickham, Vojtech Gabriel, Christopher Zdyrski, Rachel A. Allbaugh, Dipak Kumar Sahoo, Lionel Sebbag, Jonathan P. Mochel, Karin Allenspach

**Affiliations:** ^1^Department of Veterinary Clinical Sciences, Iowa State University, Ames, IA, United States; ^2^SMART Lab, Department of Biomedical Sciences, Iowa State University, Ames, IA, United States; ^3^Koret School of Veterinary Medicine, Hebrew University of Jerusalem, Rehovot, Israel; ^4^3D Health Solutions Inc., Ames, IA, United States

**Keywords:** organoid, cornea, canine, feline, stem cell, comparative ophthalmology

## Abstract

In this study, we isolated and cultured canine and feline 3D corneal organoids. Samples derived from corneal limbal epithelium from one canine and one feline patient were obtained by enucleation after euthanasia. Stem cell isolation and organoid culture were performed by culturing organoids in Matrigel. Organoids were subsequently embedded in paraffin for further characterization. The expression of key corneal epithelial and stromal cell markers in canine and feline organoids was evaluated at the mRNA level by RNA-ISH and at the protein level by immunofluorescence (IF) and immunohistochemistry (IHC), while histochemical analysis was performed on both tissues and organoids using periodic-acid Schiff (PAS), Sirius Red, Gomori's Trichrome, and Colloidal Iron stains. IF showed consistent expression of AQP1 within canine and feline organoids and tissues. P63 was present in canine tissues, canine organoids, and feline tissues, but not in feline organoids. Results from IHC staining further confirmed the primarily epithelial origin of the organoids. Canine and feline 3D corneal organoids can successfully be cultured and maintained and express epithelial and stem cell progenitor markers typical of the cornea. This novel *in vitro* model can be used in veterinary ophthalmology disease modeling, corneal drug testing, and regenerative medicine.

## Introduction

The corneal epithelium is a stratified squamous non-keratinized epithelium that comprises the outermost layer of the cornea (1). It is a critical tissue for maintaining ocular surface health by providing a barrier to microbes and toxins, anchoring the mucin layers of the tear film, and providing a smooth optical surface for light to enter the eye, among other key functions (1). Corneal epithelial cells have a high turnover rate, continuously being replenished by new epithelial cells originating from progenitors cells (limbal epithelial stem cells) in the corneoscleral junction (limbus). More specifically, limbal epithelial stem cells originate from the epithelial basal layer of the limbus, which encompasses the transitional zone between the cornea and the more peripheral bulbar conjunctiva (2). These stem cells have recently been intensively studied to investigate how regeneration of the ocular structures normally occurs in people (3–6).

Conventional 2D epithelial cell lines of the cornea are available for ophthalmic research in several species (7), although such *in vitro* tools can only model one of each specific epithelial sub-cell type. Therefore, they do not faithfully represent the complex 3D nature of the corneal epithelium and its differentiation into different cell types, as it occurs *in vivo*. Further, because conventional 2D-epithelial cell lines are immortalized, such models usually more closely resemble tumor cells than normal epithelial cells (8). For example, a human corneal epithelial cell-transformed cell line (HCE-T) was found to lack cornea-specific differentiation markers, illustrating the need for a novel and more reliable biological *in vitro* model (9).

Organoids represent mini-models of epithelial tissues and can be derived from adult epithelial stem cell populations (10, 11). Organoids can be cultured long-term and can contain multiple differentiated epithelial cells from the original tissue (12). Therefore, this technology can be used to model healthy and diseased epithelial tissues from patients and has the potential to reduce the number of animals used in drug efficacy and toxicity trials (9, 12, 13). Specifically, corneal epithelial organoids could help clinicians and scientists better characterize the pathogenesis of corneal abnormalities presumably linked to stem cell deficiency, such as pigmentary keratopathy, chronic superficial keratitis, and symblepharon (14, 15).

Induced pluripotent stem cells (iPSCs) and adult stem cells-derived organoids are two distinct approaches to generate 3D cell cultures (13). Human corneal organoids were first cultivated from iPSCs in 2017 (16, 17), and the first corneal organoids cultured from adult stem cells derived from the limbus were reported in 2020 by Higa et al. (18). Advantages of adult-stem cell-derived organoids include the possibility of seemingly limitless expansion of the culture, cell programming upon interactions with environmental triggers, as well as the high yield of differentiated cell lines that can be achieved as compared to iPSCs (12). In the 2020 study by Higa et al., adult stem-cell derived corneal organoids were successfully engrafted in a rabbit model of limbal stem cell deficiency, illustrating the clinical potential of organoid technology for the treatment of various ocular diseases through regenerative medicine approaches.

More standardized techniques and laboratory methods for the culture of adult stem-cell-derived organoids in dogs have recently been published (19). In this study, we aimed to provide proof-of concept that 3D canine and feline corneal organoids can be developed and maintained in culture. In doing so, we aimed to establish standard operating procedures for the isolation, culture, and characterization of canine and feline corneal organoids.

## Materials and methods

### Tissue harvesting

Tissues were harvested from animals euthanized for reasons unrelated to the study. Both eyes of one dog (10 years old, mixed breed, castrated male) and one cat (16 years old, domestic short hair, castrated male) were used for the experiment. Ophthalmic examination was performed before euthanasia (slit-lamp biomicroscopy, indirect ophthalmoscopy, rebound tonometry, Schirmer tear test-1, and fluorescein staining) to confirm that both eyes were free of pre-existing ocular disease. Bilateral transconjunctival enucleation was performed immediately following euthanasia of the animals. Globes were immediately submerged in Advanced Dulbecco's Modified Eagle Medium (Advanced DMEM/F12, Ref. 12634-010, Gibco) enriched with penicillin 100 μg/mL and streptomycin 100 μg/mL (Ref. 15140-122, Gibco), transported to the research facility laboratory and stored at 4°C. Limbal tissues were harvested by carefully removing the sclera, iris, and corneal endothelial tissues under a surgical microscope (WILD M691). Limbal tissues measuring approximately 2 mm in diameter were harvested with Wescott tenotomy scissors and submerged in Advanced DMEM/F12 medium. A portion of the collected tissues was also transferred to 4% formaldehyde (10% formalin) for 24 h and then changed to 70% ethanol for later paraffin embedding.

### Organoid isolation

Methods for organoid isolation and culture were based on a modified protocol established by Saxena et al. (20). Further modified from Gabriel et al. (21). Briefly, the isolated corneoscleral limbal tissue was minced and transferred to a complete chelating solution (CCS, composition provided in Supplementary Table 1). The solution was then mixed and allowed to settle at the bottom of the tube. The supernatant was replaced with fresh CCS, and the procedure was repeated four more times. Subsequently, the sample was incubated with 0.5M EDTA (Ref. 15575-038, Invitrogen) at 4°C for 10 min. The sample was then transferred to a solution CCS and fetal bovine serum (FBS, Ref. 35-010-CV, Corning) to stop the reaction. The solution was spun at 700 g for 5 min at 4°C. The supernatant was removed, replaced with Advanced DMEM/F12, and the sample was spun again using the same conditions. At last, the supernatant was removed, and the pellet was resuspended in 30 μL of Matrigel Matrix (Ref. 356231; 356230, Corning) per well. The tissue and stem cells were subsequently plated in a 24-well plate and incubated at 37°C for 20 min.

### Organoid culture

Organoids were cultured at 37°C with 5% CO_2_. The media was enriched with fibroblast growth factors 2, 7, and 10, Y-27632 ROCK inhibitor, and GSK3β (the organoid media composition is provided in Supplementary Table 1). Enriched media (500 μL/well) was changed every other day (Monday and Wednesday), with wells receiving 750 μL/well on Fridays. Cleaning refers to dissolving the Matrigel in cold Advanced DMEM/F12, centrifuging the solution at 700 g for 5 min at 4°C, and re-plating it in Matrigel. Passaging was performed by dissolving Matrigel in cold Advanced DMEM/F12, incubating organoids in TrypLE Express (Ref. 12604-021, Gibco) for 10 min, stopping the reaction using Advanced DMEM/F12, and plating the cells back in Matrigel. This step was performed after 2 weeks of initial culture for the canine organoids, and after 1 week for the feline corneal organoids. Subsequently, organoids were cryopreserved in a freezing solution (composition provided in Supplementary Table 1) and stored in liquid nitrogen for future use. A portion of the organoids was also fixed with formalin-acetic acid-alcohol (FAA, composition provided as Supplementary material) for paraffin embedding.

### RNA *in situ* hybridization

RNA-ISH (RNAScope^®^, ACD, Newark, CA) was used to characterize mRNA expression of specific molecular markers in the tissues and organoids, as previously described by Wang et al. (22). Briefly, tissues and organoids were paraffin-embedded as recommended by the manufacturer. Paired double-Z oligonucleotide probes for both canine and feline corneal samples were designed against target mRNA using custom software by the manufacturer. Probes for *in situ* hybridization included (i) N-Cadherin (epithelial stem cells), (ii) Transformation-related protein 63 (p63) (progenitor cell markers), (iii) Collagen IV (corneal and limbal epithelial basement membrane component), (iv) Leucine-Rich Repeat-containing G-Protein Coupled Receptor 5 (LGR5) (stem cell marker), (v) Aquaporin 1 (AQP1) (stromal and endothelial cell marker), and (vi) Cytokeratin 19 (CK-19) (differentiated epithelial cell marker). Further description of the probes is included in Supplementary Table 2.

The POLR2A (Polymerase II, RNA, Subunit A) probe, a general positive control used to determine if the RNA-ISH assay was successful, was used for the RNA-ISH assay. RNAscope^®^ 2.5 HD Detection Kit (Ref. 322360) was used according to the protocol provided by the manufacturer.

The slides were subsequently imaged by light microscopy (OLYMPUS BX 40), and the presence or absence of the markers was evaluated using a 40x objective. Positive control for RNA *in Situ* Hybridization Markers were run alongside sample slides. Positive control used was POLR2A (Supplementary Figure 1).

### Immunofluorescence (IF)

To deparaffinize the slides, the specimens were put in xylene twice for 10 min, then transferred to 100% alcohol twice for 1 min. Agitation was done throughout. After the last alcohol wash, slides were laid out on tissue paper for 5 min to dry. After deparaffinization, tissues and organoids underwent heat induced epitope retrieval with either Citrate buffer (pH 6) or a Tris/EDTA buffer (pH 9) using a HybEZ™ II Oven at 75°C for 2 h. After 2 h, the tray was taken out of the oven and the slides were allowed to cool with the lid off for 15 min. Once cool, the slides were rinsed in PBS twice for 2 min each, then rinsed in PBS for 10 min. Tissues and organoids were then permeabilized by incubating twice in 0.25% Triton in PBS for 10 min each. After being rinsed in PBS three times, tissues and organoids were blocked in Casein in PBS (Ref. 37528, Thermo Scientific) for 1 h at room temperature. Tissues and organoids were incubated with their primary antibody overnight at 4°C at the appropriate concentration. The next day, slides were again rinsed in PBS, a secondary antibody was added at 1:1,000 in PBS for 1 h at room temperature, and slides were rinsed again. DAPI mounting solution (Ref. ab104139, Abcam) was used to mount the slides, and after drying overnight the slides were imaged on either a Stellaris Confocal (Supplementary Figure 1) or Keyence BZ X-100 (Supplementary Figure 2) the next day. P63 (Ref. GTX102425, Genetex) was used in a concentration of 1:50 in a Citrate buffer. AQP1 (Ref. NBP1-84488, Novus Biologicals) was used in a concentration of 1:50 in a Tris/EDTA buffer.

Probes for negative controls without primary antibody were included in each experiment (Supplementary Figure 2).

### Light microscopy and immunohistochemistry (IHC)

For light microscopy, five-micron sections from paraffin embedded tissues were cut, prepared, and stained with Periodic Acid Schiff (PAS), which stains for mucopolysaccharides, mucoproteins, and glycoproteins in tissue. Tissues containing PAS positive elements (fungi, glycogen, and mucous) were used as positive control. Colloidal iron staining (solution made in-house) was used to detect acid mucins, Gomori Trichome (Kit 9176-Newcomer Supply) was used to stain for collagen, and Sirius red (solution made in-house) was used to stain collagen.

Immunohistochemistry was completed at the Department of Pathology and imaged and scanned using a Leica Aperio GT 450 Scanner. The immunohistochemical procedures used have been described previously (23). In brief, sections were deparaffinized and rehydrated using an automated system with manual antigen retrieval and blocking steps. Primary antibody, secondary antibody, horseradish peroxidase-streptavidin, and NovaRed staining were applied on the section in a sequential order followed by counterstaining. IHC was used to stain for keratinized tissue (Pan Cytokeratin antibody, Pan-CK—Agilent; Ref# M0821). Presence and absence of positive staining was evaluated using light microscopy and the cell types determined by morphology, location and staining. Tissues on positive control slides other than the target tissue were examined to ensure absence of inappropriate staining. Positive controls for Pan-CK included normal eyelid, oral mucosa, tonsil, thyroid, lung, thymus, liver, kidney, pancreas, stomach, intestine (jejunum), colon, adrenal gland, and mammary gland for each species.

Slides were reviewed by an ophthalmology resident, a board-certified ophthalmologist, a board-certified veterinary internist, and a research scientist under the supervision of a pathologist.

## Results

### Organoid isolation and culture

**Figure 1:**
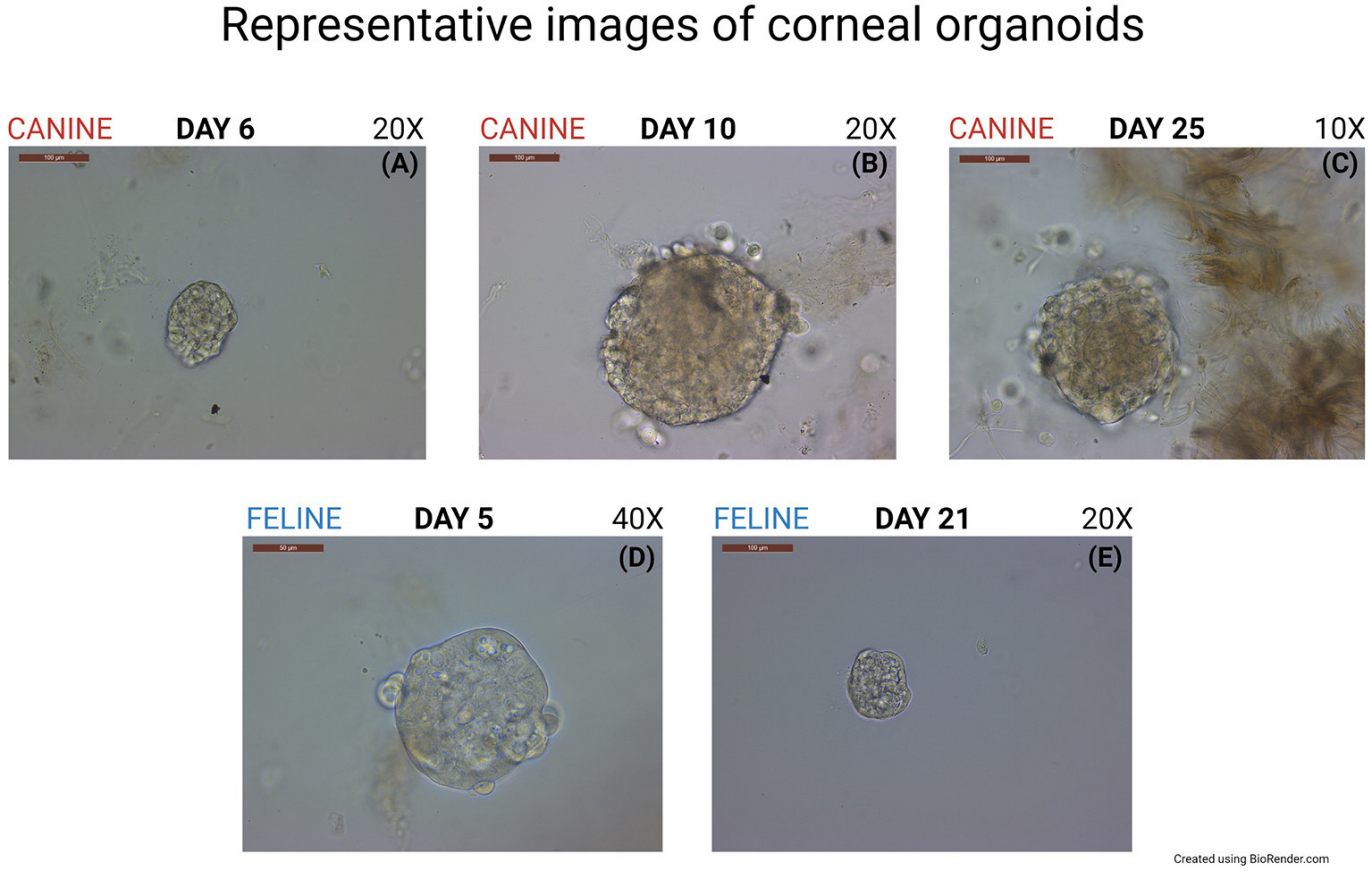
Generation of canine and feline organoids. **(A)** Representative phase- contrast microscopic images of the canine corneal organoids at passage zero-day 6 after culturing the cells, Scale bar: 100 μm. **(B)** Canine corneal organoids at passage zero-day 10 after culturing the cells, Scale bar: 100 μm. **(C)** Canine corneal organoids at passage zero-day 25 after culturing the cells, Scale bar: 100 μm. **(D)** Feline corneal organoids at passage zero-day 5 after culturing the cells, Scale bar: 50 μm. **(E)** Feline corneal organoids at passage zero-day 21 after culturing the cells, Scale bar: 100 μm.

The methods described in the present work were successfully employed for isolation and culture of canine and feline corneal organoids from adult stem cells (Figure 1). The formation of organoids was first detected on Day 4 after canine cell harvest and on Day 2 after feline cell harvest. Organoids were uniformly round and surrounded by spindle cells in the background. 3D organoids expanded in size and transformed into multilobulated structures on Day 7 for canine cells and Day 10 for feline cells.

Canine organoids were cultured for a total of 29 days. Plates were cleaned twice and passaged once to expand the organoid colonies into more wells before harvesting. Feline organoids were cultured for a total of 40 days, cleaned six times, and passaged once before harvesting. Paraffin-embedded organoids were stained by hematoxylin and eosin or left unstained to perform IHC, RNA-ISH, or IF. RNA-ISH results are summarized in Table 1.

**Table 1:** RNA *in situ* hybridization results.

**Marker**	**Canine organoid**	**Canine tissue**	**Feline organoid**	**Feline tissue**
COL4A1	Positive	Positive—limbus and epithelium	Negative	Negative
P63	Positive	Positive—epithelium and limbus	Positive	Positive—limbus and epithelium
N-Cadherin	Positive-low expression	Negative	Positive—low expression	Negative
AQP1	Positive	Positive—limbus and stroma	Positive	Positive—limbus and stroma
CK19	Negative	Positive—limbus	Positive	Positive—limbus
LGR5	Negative	Positive—epithelium	Positive—low expression	Negative

This table summarizes the results of RNA *in situ* hybridization comparison between organoids and tissue.

### Immunofluorescence (IF) and RNA *in situ* hybridization

**Figure 2:**
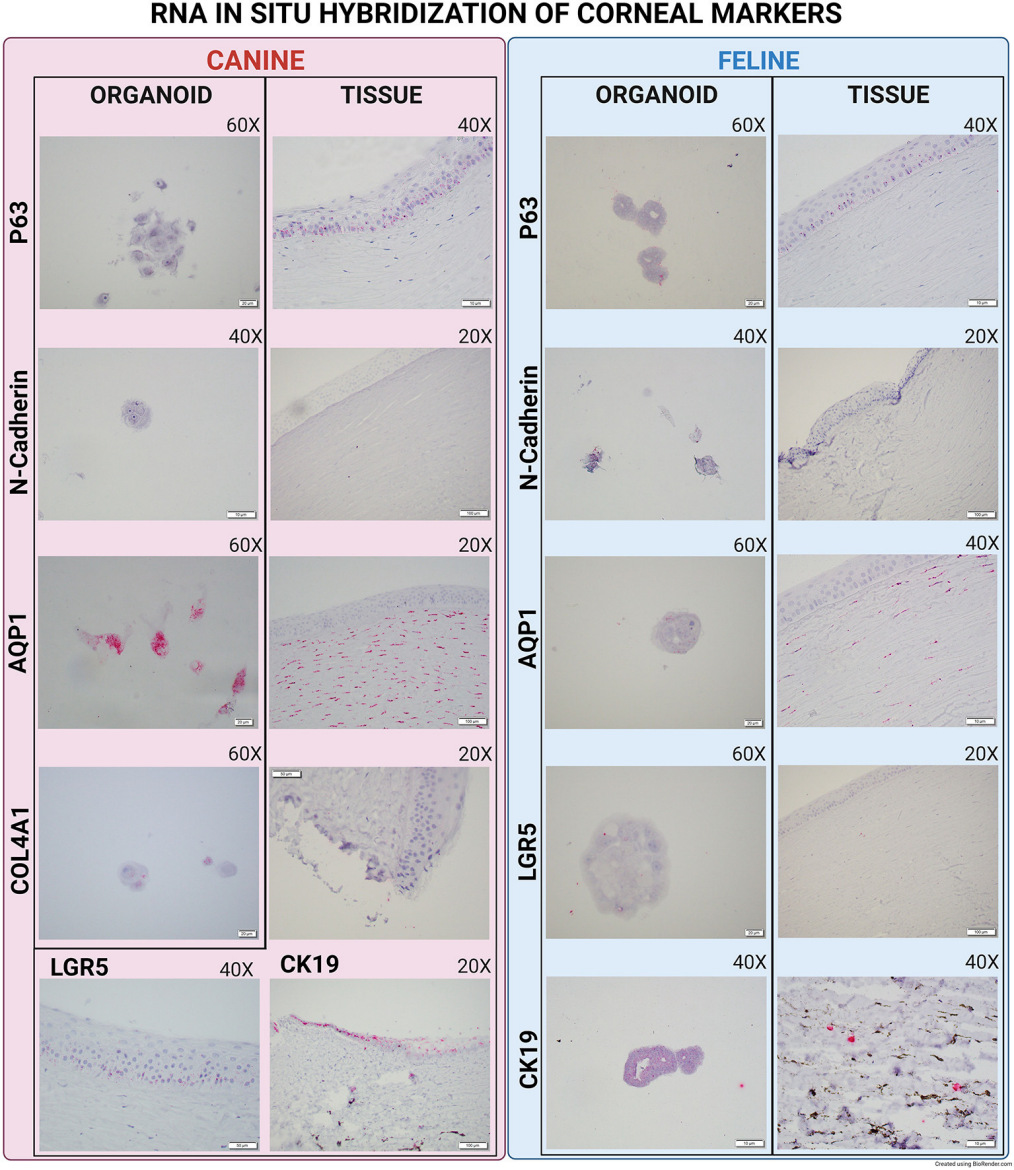
Direct compassion of RNA-ISH marker expression in corneal organoids and harvested limbal tissues of Canine and Feline organoids. Expression of various markers in canine and feline organoids and tissue. All images were evaluated for presence or absence of expression. Canine organoids and tissues were evaluated for P63, N-Cadherin, AQP1, Collagen IV, CK19, and LGR5. Feline organoids and tissues were evaluated for P63, N-Cadherin, AQP1, LGR5, and CK19. Tissue and organoid expression for each species was then compared.

Overall, Type IV collagen (COL4A1) was found to be expressed in canine organoids and the original histological sections of the limbus and corneal tissue of the same dog, whereas the marker was not expressed in feline organoids nor its corresponding tissue sections (Figure 2).

**Figure 3:**
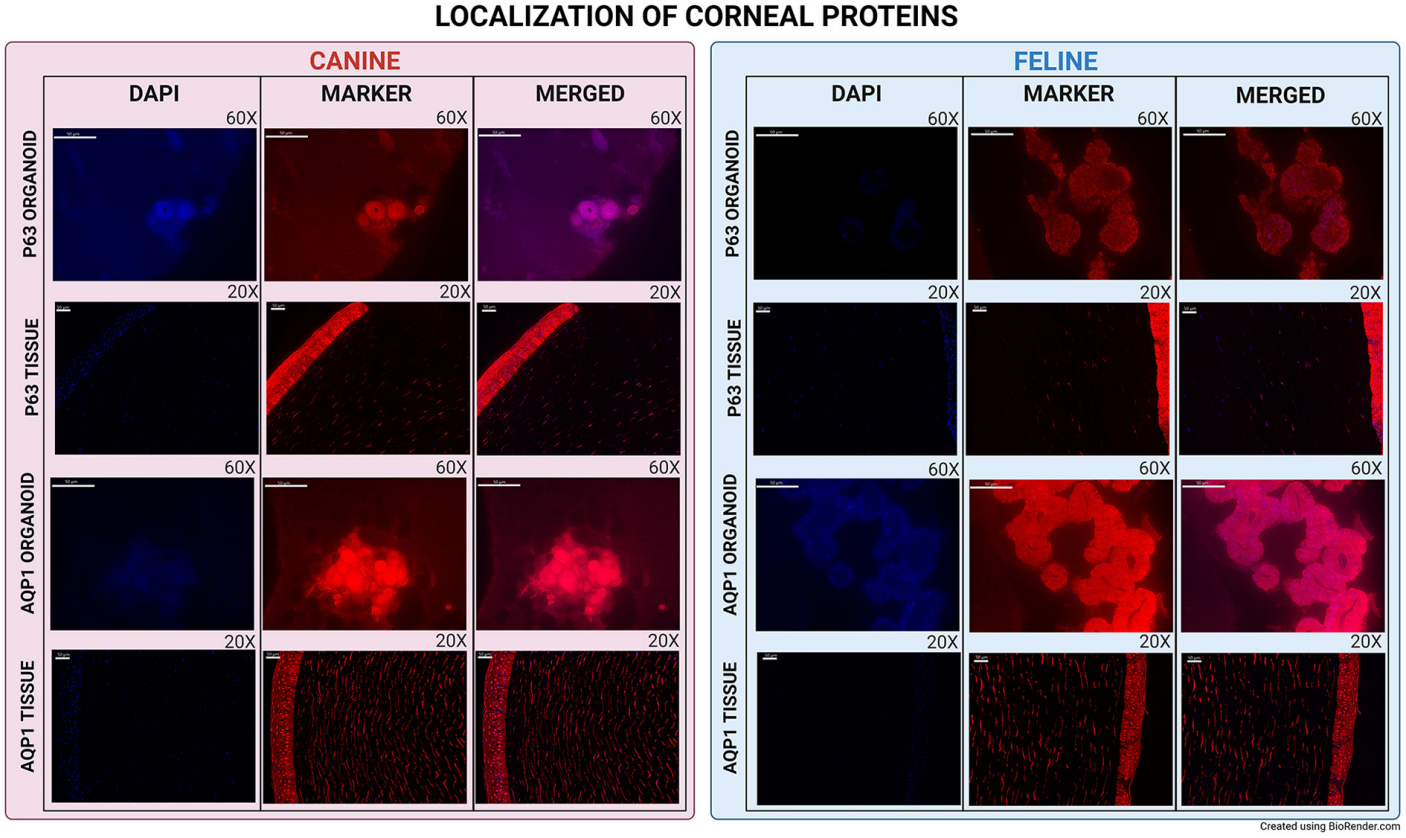
Immunofluorescence of canine and feline organoids and tissues. Immunofluorescence of P63 and AQP1 for canine and feline organoids and tissues. All images were evaluated for presence or absence of expression. Tissues were used to evaluate the efficacy of the primary antibody and as a comparison point for the organoids. Organoids pictures were taken at 60X and tissues at 20X. All scale bars are 50 μm.

P63 (Transformation-related protein 63) mRNA was expressed in organoids, corneal, and limbal tissue sections in both species. Using IF, p63 protein was expressed in canine corneal limbal epithelium and stroma tissue sections in both species, but only in canine organoids (Figure 3). N-Cadherin (Cadherin-2) showed low expression in both canine and feline organoids and was not expressed in the original tissue of either species (Figure 2). AQP1 (Aquaporin-1) was expressed in organoid samples, limbus, and stroma of tissue sections in both species at both the mRNA and the protein level using either RNA-ISH or IF (Figure 3).

**Figure 4:**
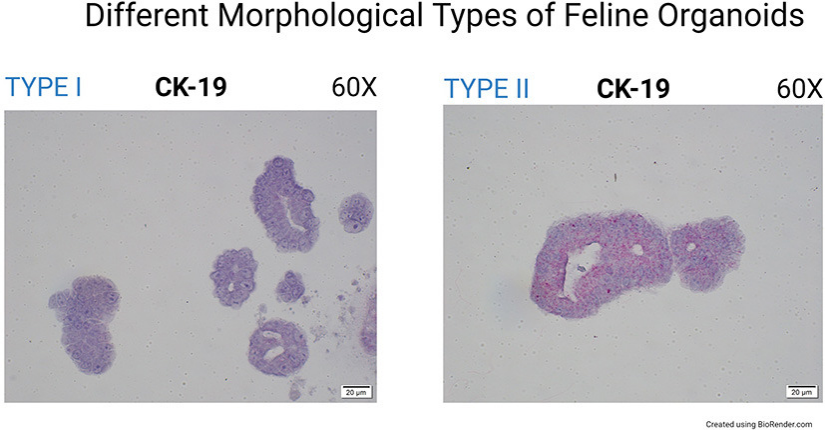
Different morphological types of feline organoids (60X). The image on the left shows highly cellular organoids (*type I*). Organoids in the right image represent a less cellular type of organoids (*type II*). These two organoids show markedly different expression of CK19.

CK19 (Cytokeratin-19 or Keratin-19) mRNA was not found in canine organoids but was expressed in the corresponding tissue section at the limbus (Figure 2). The expression of CK19 was detected in feline organoids and the limbus of the feline tissue sections (Figure 2). Interestingly, two organoid phenotypes were observed in the feline corneal organoid cultures. Some organoids (*type 1*) were only slightly positive for CK19 and composed of a bi/tri-nucleated cellular structure with perinuclear clearing (Figure 4). Another population of feline 3D corneal organoids (*type 2*) was highly positive for CK19, presenting with less cellularity and a more heterogeneous morphology (Figure 4).

Finally, LGR5 (Leucine-Rich Repeat-containing G-Protein Coupled Receptor 5) was not expressed in canine organoids but was expressed in the epithelium of the canine cornea tissue samples. LGR5 was weakly expressed in feline organoids, but not in any layers of the feline corneal tissue samples (Figure 2).

### Light microscopy and immunohistochemistry (IHC)

**Figure 5:**
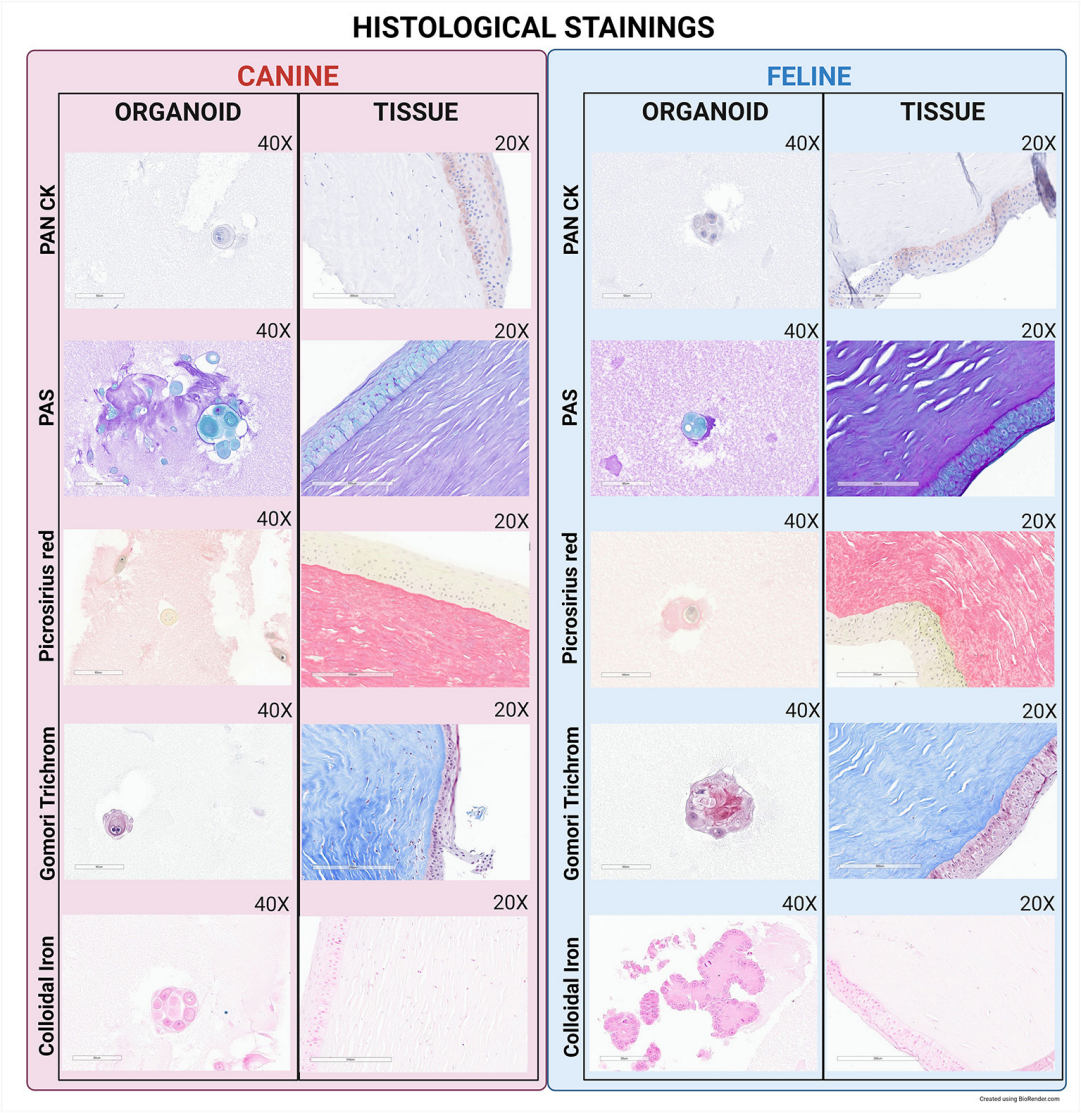
Histological and IHC staining of canine and feline organoids. To further evaluate expression patterns of organoids compared to the parent tissue, additional staining was completed, including PAN CK, PAS, Picrosirius Red, Gomori Trichrome, and Colloidal Iron. Organoid images were taken at 40X with a 60 μm scale bar while tissue images were taken at 20X with a 200 μm scale bar.

PAS was positive for both tissue samples and organoids (Figure 5). Colloidal iron was positive in both canine and feline tissues and was seen to be weakly positive in organoids (Figure 5). The presence of collagen was interpreted through Gomori's Trichrome and positive in both canine and feline tissues; however, it was absent in both canine and feline organoids (Figure 5). Sirius red stains collagen and amyloid connective tissue and was positive in the tissues, but negative in the organoids of either species (Figure 5). Pan-CK labeling was detected in epithelial cells in tissue samples and organoids of both species (Figure 5).

## Discussion

This study demonstrates for the first time, that corneal organoid cell lines can be successfully established from canine and feline limbal tissue sections. Furthermore, 3D the corneal organoids were successfully established from adult stem cells from older animals (10-year-old dog and 16-year-old cat).

3D canine and feline organoids were morphologically comparable to the human corneal organoids recently established from adult stem cells by Higa et al. (18). Of note, some of the stem cells in the canine and feline organoid cultures started to grow in clusters rather than forming the characteristic spheroid-like structures reported in previously published literature (24). The expansion of these cells was terminated after 3 days. Given this unusual cell organization in some corneal organoids we hypothesize that some stem cells could not be expanded into fully formed organoids. This issue could be secondary to suboptimal conditions for corneal organoid expansion due to the lack of specific media components. Higa et al. enhanced their corneal organoid media with tri-iodothyronine, isoproterenol hydrochloride, human recombinant insulin, and hydrocortisone (18). The addition of these components could therefore potentially have improved the expansion of these single stem cells into more advanced corneal organoid cultures of dogs and cats which could be further investigated in future studies (18).

N-cadherin was previously identified in the human limbal epithelial stem cell niche as a putative marker of epithelial progenitor cells (2). The expression of N-cadherin in canine and feline limbal tissues, however, was not confirmed in the present study. In a recent study in human corneal organoids, N-cadherin was sporadically expressed in limbal basal epithelial cells at low levels (18). This latter observation is consistent with our results in canine and feline organoids as we found weak expression of N-Cadherin mRNA in corneal organoids of both species. Notara et al. observed a sharp reduction of limbal epithelial crypts housing limbal epithelial stem cells in people older than 60 years (25). Since the animals used for tissue harvest in our study were middle-aged to older, the expression of the N-cadherin marker in the tissues could have been lower due to reduced numbers of stem cells present in the corneas of these older animals. Higa et al. showed how niche-like cells underneath the limbal epithelial Descemet's membrane directly interact with N-cadherin positive cells (26). We therefore hypothesize that activation and proliferation of the stem cells during isolation and cultivation of the organoids could have stimulated differentiation and proliferation of niche-like cells and N-cadherin positive cells, even though this marker was not found in the tissues they were derived from.

Using RNA-ISH, we were able to show that p63 protein was expressed in organoids, limbus, and corneal epithelial tissues of both species. p63 promotes expansion of limbal progenitor cells and plays a pivotal role in retaining the cornea's proliferative capacity (27). The presence of p63 in canine and feline corneal epithelial organoids in our study indicates the presence of limbal epithelial progenitor cells (28). This marker has also previously been found to be upregulated after corneal injury (28). It is possible that the expression of p63 would be altered in corneal organoids derived from animals that had sustained corneal damage, but this assumption will need to be validated in future studies. Next, we additionally used IF to detect protein expression of p63, which showed that dog and cat corneal tissues were positive for p63 in the corneal basal epithelium with some staining also present in the corneal stroma. This finding is consistent with a previous study by Morita et al. which found positive staining of p63 in the limbal basal layer of canine eyes on IHC and PCR (28). Previous reports have found variable expression of p63 within the cornea, with some of them detecting expression in the basal layer of limbal epithelium and not in the corneal epithelium in humans, while others showed strong expression in human limbal basal cells and the central cornea (29, 30). Our results and the results from previous findings, therefore, suggest that p63 expression may be related to the proliferative potential of corneal epithelial cells in dogs (31, 32).

Collagen IV is normally present in the basement membrane and the anterior epithelium of the cornea and plays an important role in maintaining the limbal epithelial phenotype in the epithelial sheets with structural support (33, 34). Collagen IV was present in the tissue surrounding individual cells forming canine corneal epithelial organoids. There were also spindle-like mesenchymal cells present in our culture. This latter finding is consistent with observations made in cultures of human corneal organoids, where mesenchymal cells are frequently found in the background of organoid cultures (18). These stromal cells (keratocytes) likely have the ability to induce supportive tissue growth, as well as to produce growth factors for the epithelial organoids in culture (35). As expected, canine tissue sections and organoids showed high expression of Collagen IV in both limbal and epithelial regions. This suggests that organoids derived from one donor could, in the future, supply stratified epithelial sheets containing progenitor cells for multiple recipients for regenerative medicine purposes.

On the contrary, feline organoids and tissues did not express Collagen IV. To the author's knowledge, no studies investigating the presence of Collagen IV in feline corneal tissues have been performed to date. We hypothesize that this finding could indicate that feline corneal epithelial cells do not normally express the Collagen IV marker, contrary to human and dog corneal epithelial cells (33, 36, 37).

Since probes had to be specifically designed and there is no published data on the presence of collagen IV in feline corneal tissues, we cannot rule out that the RNA probe for Collagen IV used in this study did not work for the feline species, though it did work for the positive control.

Since we were unable to confirm expression of collagen IV in the cat cornea using RNA-ISH, we also used Gomori Trichome staining, which typically stains collagen fibers and muscle which was positive in the feline corneal stroma.

LGR5 serves as a marker for corneal stem cells located in the limbal epithelial crypts (38). In the canine tissue, LGR5 was positive in tissues but was not expressed in organoids. This observation could be explained by the old age of the canine donor and the presence of a higher percentage of stem cells in the tissue compared to the organoids (25). There was no expression of LGR5 in feline tissues, but feline organoids expressed the marker in small quantities. These results could be explained by low affinity of the probe to the mRNA, or that LGR5 is not expressed in the feline cornea. To our knowledge, no data is available regarding LGR5 expression in the feline cornea, and further investigation is warranted.

AQP1 is a mesenchymal marker normally expressed in corneal endothelial cells, stromal keratocytes, and the limbal area in mice, rats, and humans (26, 39–41). This observation is consistent with the findings from our study in dogs and cats, as AQP1 was detected in the limbus and stromal areas of the tissue sections in both RNA-ISH and IF. Interestingly, this marker was also positive in organoids from both species, suggesting that some cells in the organoid culture may have the ability to differentiate into keratinocytes or endothelial cells.

CKs have been used as markers for cornea-specific epithelial differentiation and stem cell characterization located in the limbus (14, 42).

CK19 was not detected in canine organoids but was found in several of the feline organoids. In a previous equine study, the expression of CK19 was not limited to stem cells, and CK19 was therefore considered a marker of epithelial differentiation, rather than an explicit stem cell marker, in this species (43). The authors of the latter study concluded that the presence of CK19 and the absence of Cytokeratin 3 (CK3) determines the presence of stem cells in the limbus. CK3 expression was not evaluated in the present study, but this could represent another marker to be investigated in the future. In humans, CK19 expression is limited to the corneal periphery, limbus, and perilimbal conjunctiva (44). This observation is consistent with our findings in dogs and cats, as CK19 expression was limited to the limbal area of our tissue samples. The difference in expression levels of CK19 in organoids of different morphology (*type 2 organoids*) that we describe here warrants further investigation in future studies.

IHC was used to identify cell surface expression of epithelial cells and their lineage proteins. CKs are water-soluble proteins that are characteristic for epithelial cells (45). Immunohistochemistry staining revealed positive staining for Pan-CK, indicating that organoids are likely of epithelial origin, specifically originating from the basement membrane. Positive staining for PAS in the epithelial membrane of the organoids indicated that they could differentiate into specialized epithelial cells. Sirius Red staining, while positive in the tissues, was negative in the organoids, showing a lack of connective tissue cells in the organoids (46). While positively expressed in both canine and feline tissues, organoids were negative for Gomori's Trichrome staining (which stains organized collagen sheets) and only weakly positive for Colloidal Iron (which stains primitive collagen). Therefore, primitive collagen is likely present in the organoids of our study, but it is not organized into fibers and collagen sheets (47).

Since stem cells are responsible for the continuous re-population of the corneal epithelium, diseases that induce limbal stem cell deficiency (LSCD) result in corneal conjunctivalization and neovascularization, corneal scarring, and chronic inflammation (1). In human medicine, selected congenital and acquired limbal stem cell deficiencies include aniridia, various forms of keratitis, dyskeratosis congenita, epidermal dysplasia, chemical and thermal burns, ocular surgeries involving the limbal region, use of contact lenses, ocular surface inflammatory diseases, and Stevens-Johnson syndrome (7).

Organoids reliably represent epithelial tissue microanatomy of their original organ and replicate both physiologic or pathophysiologic processes of the donor tissue (48). Similar to humans, several ocular diseases in companion animals may also be linked to LSCD, including canine herpes virus-1 keratitis, symblepharon in cats secondary to feline herpesvirus-1 infection, chronic ocular irritation, and immune-mediated conditions like keratoconjunctivitis sicca, chronic superficial keratitis (pannus), pigmentary keratopathy, and aniridia (15). 3D organoids derived from animals displaying various diseases of the cornea could enable the *in vitro* study of such corneal dysfunction, which has so far been impossible in companion animals using traditional 2D cell lines. Other novel applications of the corneal organoids described in this study could include drug testing and regenerative medicine approaches (41, 49).

This proof-of-concept preliminary study has several limitations. In particular, the single donor for each species does not allow for inter-animal variations to be studied. Further research should utilize additional corneal samples to confirm the findings of our study. Moreover, the use of younger animals may result in differences in culture efficiency and cellular differentiation of the organoids. Furthermore, the influence of breed and sex on organoid culture and differentiation could not be studied with the limited number of available donors.

Despite the fact that RNA-scope probes are bioinformatically designed to selectively target mRNA and can overcome the sensitivity and specificity constraints often associated with antibodies used for IHC, fewer animal research than human studies have utilized this technology, and genomes for some species, such as the feline, have not been sequenced as thoroughly as others. This fact may account for some oligonucleotide probes not binding as would be predicted by their DNA sequence.

In conclusion, 3D corneal epithelial organoids were established from adult stem cells harvested from canine and feline donors. Initial molecular characterization of 3D organoids using RNA -ISH, IHC and IF further confirmed the expression of specific markers of corneal epithelial and stromal cells in the organoids. This novel and innovative *in vitro* model system could be refined and be used in veterinary ophthalmology for disease modeling, corneal drug testing, and regenerative medicine applications.

## Data availability statement

The raw data supporting the conclusions of this article will be made available by the authors, without undue reservation.

## Ethics statement

Ethical review and approval was not required for the animal study because the eyes were taken post-mortem. Animals were referred to Iowa State University by the referring veterinarian to a service other than ophthalmology. Tissues were harvested from animals euthanized at our facility for reasons unrelated to eye issues. A euthanasia protocol combining intravenous propofol and pentobarbital was used, doses depended on the body weight. Written informed consent was obtained from the owners for the participation of their animals in this study.

## Author contributions

All authors listed have made a substantial, direct, and intellectual contribution to the work and approved it for publication.

## Conflict of interest

Author KA is a co-founder of 3D Health Solutions. She serves as a consultant for Ceva Animal Health, Bioiberica, LifeDiagnostics, Antech Diagnostics, Deerland Probiotics, and Mars. Author JM is a co-founder of LifEngine Animal Health and 3D Health Solutions. He serves as a consultant for Ceva Animal Health and Ethos Animal Health. The remaining authors declare that the research was conducted in the absence of any commercial or financial relationships that could be construed as a potential conflict of interest.

## Publisher's note

All claims expressed in this article are solely those of the authors and do not necessarily represent those of their affiliated organizations, or those of the publisher, the editors and the reviewers. Any product that may be evaluated in this article, or claim that may be made by its manufacturer, is not guaranteed or endorsed by the publisher.
