# Supplementary material for: Culture and characterization of canine and feline corneal epithelial organoids: A new tool for the study and treatment of corneal diseases
Source: Front Vet Sci. 2022 Nov 4;9:1050467. doi: 10.3389/fvets.2022.1050467 (PMC9672346; doi:10.3389/fvets.2022.1050467)
Supplement: Supplementary file 1 [file Data_Sheet_1.docx]

### Supplementary Table 1 – Composition of organoid and freezing media, FAA, and CCS

| **Complete Chelating Solution (CCS) Composition** | **Distributor** | **Reference number** | **V/V % or final concentration** |
| --- | --- | --- | --- |
| Incomplete chelation solution | NA | NA | 20% |
| Sterile MilliQ H2O | NA | NA | 80% |
| DTT | Promega | V3151 | 520μM |
| Pen Strep | Gibco | 15140-122 | Pen: 196 U/mL; Strep 196 ug/mL |
| **Organoid Media Composition** | **Distributor** | **Reference Number** | **Final Concentration** |
| Advanced DMEM/F12 | Gibco | 12634-010 | NA |
| FBS | Corning | 35-010-CV | 8% |
| Glutamax | Gibco | 35050-061 | 2mM |
| HEPES | VWR Life Science | J848-500ML | 10mM |
| Primocin | InvivoGen | ant-pm-1 | 100 µg/mL |
| B27 supplement | Gibco | 17504-044 | 1x |
| N2 supplement | Gibco | 17502-048 | 1x |
| N-Acetyl-L-cysteine | Sigma | A9165-25G | 1mM |
| Murine EGF | PeproTech | 315-09-1MG | 50 ng/mL |
| Murine Noggin | PeproTech | 250-38-250UG | 100 ng/mL |
| Human R-Spondin-1 | PeproTech | 120-38-500UG | 500 ng/mL |
| Murine Wnt-3a | PeproTech | 315-20-10UG | 100 ng/mL |
| [Leu^15^]-Gastrin I human | Sigma | G9145-.5MG | 10 nM |
| Nicotinamide | Sigma | N0636-100G | 10 mM |
| A-83-01 | PeproTech | 9094360 | 500nM |
| SB202190 (P38 inhibitor) | Sigma | S7067-25MG | 50 µM |
| TMS (trimethoprim sulfate) | Sigma | T7883-5G | 10µg/mL |
| **Freezing Media Composition** | **Distributor** | **Reference number** | **V/V percent** |
| Organoid media and ROCK inhibitor | NA | NA | 50% |
| FBS | Corning | 35-010-CV | 40% |
| Dimethyl Sulfoxide (DMSO) | Fisher Chemicals | D128-500 | 10% |
| **FAA composition** | **Distributor** | **Reference number** | **V/V percent** |
| Ethanol (100%) | NA | NA | 50% |
| Acetic Acid, Glacial | Fisher Chemical | A38-500 | 5% |
| Formaldehyde (37%) | Fisher Chemical | F79P-4 | 10% |
| Distilled water | NA | NA | 35% |
| **Incomplete Chelating Solution (ICS) Composition** | **Distributor** | **Reference Number** | **Final Concentration** |
| 500 ml MilliQ H2O | NA | NA | NA |
| 2.49 g Na_2_HPO_4_-2H_2_O | Sigma | S5136-100G | 4.98 mg/mL |
| 2.7 g KH_2_PO_4_ | Sigma | P5655-100G | 5.4 mg/mL |
| 14 g NaCl | Fisher Chemical | S271-500 | 28 mg/mL |
| 0.3 g KCl | Fisher Chemical | P217-500 | 0.6 mg/mL |
| 37.5 g Sucrose | Fisher Chemical | S5-500 | 75 mg/mL |
| 25 g D-Sorbitol | Fisher Chemical | BP439-500 | 50 mg/mL |
| **Additional components** | **Distributor** | **Reference Number** | **Final Concentration** |
| Human FGF-basic (154 a.a.) | PeproTech | 100-18B-10UG | 12.5 ng/mL |
| Human KGF (FGF-7) | PeproTech | 100-19-10UG | 25 ng/mL |
| Human FGF-10 | PeproTech | 100-26 | 100 ng/mL |
| ROCK inhibitor (Y-27632) | EMD Millipore Corp. | SCM 075 | 10 µM |
| Stemolecule CHIR99021 (GSK3β) | Reprocell | 04-0004-base | 2.5 µM |

Composition of the organoid media, formalin-acetic acid-alcohol, incomplete chelating solution, and complete chelating solution with distributor and reference number information.

### Supplementary Table 2 - RNAScope probes, markers, and their significance

| **Target Marker** | **Abbr.** | **Significance** | **Species** | **Probe** | **Target Region** | **Reference** |
| --- | --- | --- | --- | --- | --- | --- |
| Type IV collagen | COL4A1 | Part of the basement membrane of corneal epithelium | Canine | Cl-COL4A1-C1 | 92-1389 | 1079491-C1 |
|  |  |  | Feline | Fc-COL4A1-C1 | 506-1754 | 1079411-C1 |
| Transformation-related protein 63 | P63 | Stem cell proliferation | Canine | CI-TP63-C1 | 519-1690 | 1079081-C1 |
|  |  |  | Feline | Fc-TP63-C1 | 54-1265 | 1079041-C1 |
| N-Cadherin | N-Cad | Epithelial progenitor cell | Canine | Cl-CDH2-C1 | 391-1388 | 1079011-C1 |
|  |  |  | Feline | Fc-CDH12-C1 | 1547-2516 | 1079031-C1 |
| Aquaporin 1 | AQP1 | Stroma, endothelium | Canine | Cl-AQP1 | 3-869 | 509941 |
|  |  |  | Feline | Fc-AQP1-C1 | 2-1141 | 1079101-C1 |
| Cytokeratin 19 | CK19 | Peripheral cornea, limbus, and perilimbal conjunctiva | Canine | Cl-KRT19 | 401-1539 | 877521 |
|  |  |  | Feline | Fc-KRT19-C1 | 2-1409 | 1079111-C1 |
| Leucine-Rich Repeat-containing G-Protein Coupled Receptor 5 | LGR5 | Stem cell | Canine | Cl-LGR5 | 517-1506 | 405651 |
|  |  |  | Feline | Fc-LGR5-C1 | 722-1715 | 1079091-C1 |
| RNA Polymerase II Subunit A | POLR2A | Control | Canine | Cl-Polr2a | 1846-2924 | 310981 |

Probes used for RNA *in situ hybridization* (RNAScope by ACD, Newark, CA). It also includes a target region of the probe and its significance.
