# Supplementary figures and images for: Culture and characterization of canine and feline corneal epithelial organoids: A new tool for the study and treatment of corneal diseases
Source: Front Vet Sci. 2022 Nov 4;9:1050467. doi: 10.3389/fvets.2022.1050467 (PMC9672346; doi:10.3389/fvets.2022.1050467)

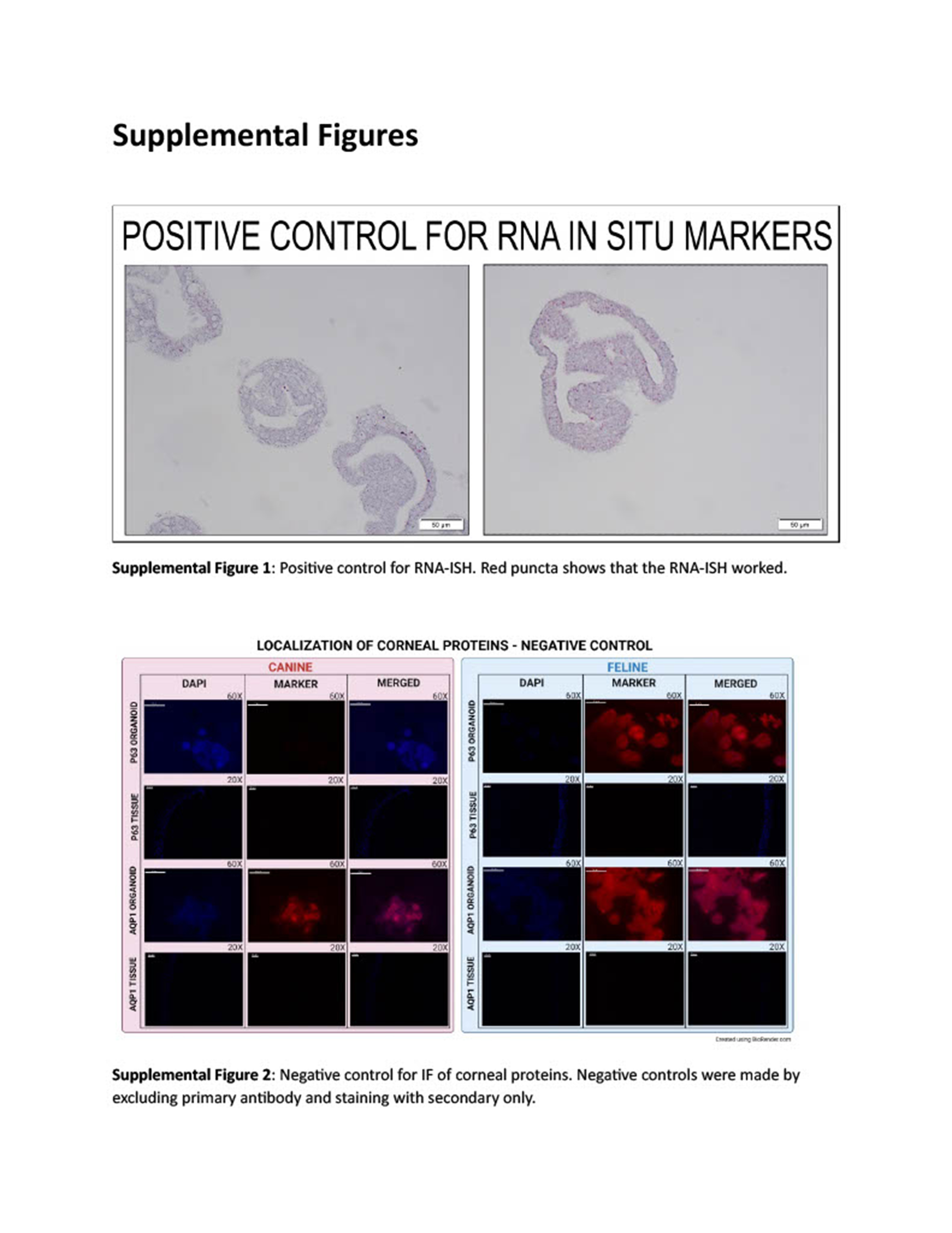

Supplement: Supplementary file 2 [file Image_1.JPEG]
